# A Mode of Preventing Pitting in Small-Pox

**Published:** 1863-08

**Authors:** R. B. Painter


### A MODE OF PREVENTING PITTING IN SMALL-POX.

I am desirous of adding my testimony in favor of a mode of preventing pitting in small-pox, not, I believe, in general use, and which, though spoken of in "Wilson on Skin Diseases," is either not mentioned or not laid stress on in our works on the general practice of physic.

I allude to the Arabian plan of pricking the pocks. I have tried it many times, and have never been disappointed in the result. Three of the cases especially abide in my memory, in which the patients were very fair, and two of them very pretty, and who all retained their fairness and beauty without a vestige of a scar.

My practice is to watch the progress of the papules, and on the fourth or fifth day, when I think the vesicles have nearly attained their full size, and *before they become pustular*, I *cut off* the apex of each vesicle with a lancet; for I find it is not sufficient to merely prick the vesicle slightly, or the exuding lymph will dry and seal the vesicle, which may thus go on to the formation of pus. This procedure will not cause the least pain if done with a sharp lancet, and a light and steady hand—the summit of the vesicle only being cut, and the flat of the instrument held on the same plane as the skin. Having opened all the pocks, I let the patient continue to lie on his back, and place a small poultice (without a rag) on such parts as are much inflamed. When these little poultices have been on an hour or so, they should be removed, the places lightly sponged, and covered with sweet oil by means of a camel's-hair brush. On the following day, if the pocks are inflamed, and matter forming beneath the crusts, I open them, and poultice again. In this way the inflammation, suppuration, and ulceration or sloughing of the skin beneath the pock are cut short, and a scar prevented.

In the last case I had, the eruption was very abundant, and confluent in places. Still I confess I have happily had no really bad confluent case since I have used this treatment, though I had much experience of such in 1848; and I think it probable that if I had a case of low type where the eruption was flattened, I should not prick the vesicles till I had by stimulating the patient got the pocks to project more fully, or I should fear that the excitement and irritation of the operation might depress the invalid; and it is even possible that the small discharge of fluid from the cuts might drain away a fraction of the strength so desirable to retain. I should not be deterred, however, in a quite confluent case if the eruption stood well out, and I think it as rational to try and cut short the inflammation of the skin in this disease as to make incisions for prevention of suppuration and sloughing in cellulitis and other affections.

An advantage of this instrument over that of the application of nitrate of silver is, that it does not cause pain or increase the febrile action, nor have I found it in any way interfere with favorable progress.

It may be objected that the process is tedious, and exposes the operator to the risk of contagion; but by getting behind the patient as much as possible, and avoiding his breath, the latter danger may be lessened; or if the necessary time cannot be spared, I see no objection to an intelligent nurse being trusted, after instruction, to perform so trivial an operation.

I believe those who try this method will find it far superior to the use of the mask or unctuous preparations, though it may be combined with the plan of covering the face.

The application of a solution of caoutchouc in chloroform, lately recommended, I have not tried; but I imagine it must produce a most unpleasant feeling of constriction, and cannot certainly be more effectual than the foregoing in preventing disfigurement.—(R. B. Painter,)—*London Lancet.*

